# Association of macronutrient quality at dinner versus breakfast with body composition in adults living in Tehran: results from a cross-sectional study

**DOI:** 10.1186/s40795-026-01382-5

**Published:** 2026-06-12

**Authors:** Shadi Ghaemi, Reyhane Norouziasl, Negar Bafkar, Bahareh Jabbarzadeh Ganjeh, Kourosh Djafarian, Alireza Bahrami, Sakineh Shab-Bidar

**Affiliations:** 1 Department of Community Nutrition, School of Nutritional Sciences and Dietetics, Tehran University of Medical Sciences (TUMS), Tehran, Iran; 2 Sports Medicine Research Center, Neuroscience Institute, Tehran University of Medical Sciences, Tehran, Iran; 3 Lifestyle Medicine Department, Medical Laser Research Center, ACECR, Tehran, Iran; 4 Health Economics and Health Technology Assessment, School of Health and Wellbeing, University of Glasgow, Glasgow, UK

**Keywords:** Meal timing, Macronutrient quality, Body composition, Obesity, Metabolic health, Nutritional epidemiology

## Abstract

**Background:**

We aimed to examine the relationship between the quality of macronutrients consumed at dinner versus breakfast and their effects on body composition among Iranian adults.

**Methods:**

A cross-sectional study was conducted among 450 Iranian adults (aged 20–59). Anthropometric and body composition measures, including BMI, body fat percentage, fat-free mass, muscle mass, and waist-to-hip ratio, were assessed. Dietary data were collected using three 24-hour recalls, categorizing macronutrient quality (carbohydrates, fats, and proteins). ANCOVA was used to compare anthropometric and body composition measures across macronutrient tertiles for dinner versus breakfast. Non-linear associations were analyzed using restricted cubic splines.

**Results:**

According to our findings, higher-quality carbohydrate intake at dinner versus breakfast showed a significant inverted U-shaped association with BMI. Conversely, saturated fat intake at dinner versus breakfast increased BMI in a U-shaped relationship, while linearly reducing muscle mass and fat-free mass. Unsaturated fat intake at dinner compared to breakfast showed a U-shaped association with BMI. Moreover, animal protein intake at dinner versus breakfast slightly increased muscle mass and fat-free mass in a reverse U-shaped pattern. However, higher plant protein intake at dinner versus breakfast reduced BMI linearly and decreased fat-free mass and muscle mass.

**Conclusions:**

Higher intake of high-quality carbohydrates, unsaturated fats, and plant-based proteins at dinner versus breakfast may improve body composition, while increased consumption of saturated fats and animal proteins at dinner versus breakfast may worsen central adiposity and BMI.

## Background

 In recent decades, lifestyle changes have led to a significant rise in the prevalence of obesity around the world [1]. According to the World Health Organization, the global rate of obesity has tripled from 1975 to 2016 [2]. More than 1.9 billion adults aged 18 and over are classified as overweight, which accounts for 39% of men and 40% of women. Among them, 650 million individuals are considered obese, representing 11% of men and 15% of women [3–5]. A systematic review conducted in Iran found that the prevalence of overweight individuals in the Iranian population ranges from 27% to 38.5%, while obesity rates vary between 12.6% and 25.9% [6]. Obesity is a major risk factor linked to various multi-organ disorders, including metabolic syndrome, cardiovascular diseases, type 2 diabetes, obstructive sleep apnea, cancer, and reproductive and endocrine disorders [3].

Previous studies indicated that macronutrient quality significantly impacts body composition and obesity [7–9]. Findings from most prior research on carbohydrate quality suggested that diets with a low glycemic index (GI) result in reduced insulin response and increased body fat oxidation. These findings indicated that a low-GI diet may be an essential strategy for preventing and managing obesity and related chronic diseases through its effects on body composition and fat oxidation [10]. Another study demonstrated that higher dietary quality was associated with reduced body fat, healthy fat distribution, and increased lean mass [11]. Recently, a study showed that improving protein quality without increasing total energy intake preserved lean mass relatively and further reduced body fat [12].

Recently, studies have focused on the relationship between food timing, known as chrono-nutrition, and body composition. Results from a systematic review indicated that individuals with evening chronotypes are more likely to experience overweight and metabolic health issues compared to those with morning chronotypes [13]. Additionally, a cross-sectional study found that later sleep times, delayed morning and evening meals, and increased food intake at night were associated with higher body fat percentages [14]. Furthermore, a study in China revealed that consuming more energy, protein, and fat at dinner compared to breakfast was linked to a higher risk of type 2 diabetes [15].

Several studies have reported an association between regular breakfast consumption and a lower risk of metabolic risk factors [16–19]. Other research connects regular breakfast intake to lower rates of obesity and being overweight [20], while increased food consumption in the evening is associated with higher rates of obesity and anthropometric measurements [21]. Recent findings suggested that consuming a significant portion of daily energy intake shortly after waking up, rather than closer to bedtime, is linked to a lower body mass index (BMI); however, this relationship may differ based on chronotype. Additionally, data indicated a clear connection between the timing of carbohydrate and protein intake and obesity [22] .

However, despite studies on the importance of meal timing, energy distribution, and macronutrient intake across meals with obesity and metabolic disorders, there remains limited understanding of whether the quality of macronutrients consumed at different meals is associated with changes in body composition. Therefore, the present study aims to examine the relationship between the quality of macronutrients consumed at dinner compared to breakfast and body composition in a sample of Iranian adults.

## Methods

### Study design and participants

This cross-sectional study was conducted in 2024 among adults aged 20–59 years who were referred to healthcare centers across Tehran, Iran. Initially, 556 men and women were assessed for eligibility. Participants were included if they were apparently healthy, resided in Tehran, were willing to participate, and provided written informed consent. Eligibility was determined based on predefined inclusion and exclusion criteria.

Participants were excluded if they had any diagnosed chronic conditions, including renal, hepatic, pulmonary, cardiovascular disorders, diabetes, or hormonal diseases; if they were pregnant or lactating; or if they reported the use of medications or dietary supplements that could influence metabolic or dietary outcomes, such as weight-loss agents, hormonal therapies, sedatives, or thermogenic supplements (e.g., caffeine, green tea extracts, or conjugated linoleic acid). Individuals with active infectious or inflammatory conditions were also excluded. In addition, participants engaged in rotating shift work or permanent night-shift schedules were excluded due to the potential impact of circadian misalignment on dietary intake and meal timing patterns.

Of the initially screened individuals, 51 were excluded based on medical and medication-related criteria, 6 due to shift-work schedules, and 49 due to incomplete questionnaires or missing essential dietary, anthropometric, or covariate data. Accordingly, 450 eligible participants completed all study procedures and were initially enrolled. Following further quality control, 6 participants were excluded due to implausible energy intake reporting (under- or over-reporting), resulting in a final analytical sample of 444 participants.

Participants were recruited using a convenience sampling method from multiple healthcare centers located in different districts of Tehran. To enhance representativeness and reduce selection bias, centers were selected from areas reflecting diverse socioeconomic backgrounds. Recruitment was conducted at different times of the day to capture individuals with varying daily routines and occupational patterns. Furthermore, all eligible adults within the target age range attending the centers during the recruitment period were invited to participate, regardless of their health status or dietary habits, provided they met the study criteria.

### Anthropometric and body composition assessments

Anthropometric and body composition assessments were performed by trained nutrition professionals following standardized measurement protocols to minimize inter-observer variation and measurement error. All measurements were conducted under supervision by trained staff members rather than by a single assessor. Height was measured without shoes using a stadiometer (Seca, Germany) to an accuracy of 0.1 cm. Weight was recorded to the nearest 0.1 kg using a digital scale (Seca 808, Germany) [23]. BMI was calculated as weight in kilograms divided by height in meters squared (kg/m²) [23]. Waist and hip circumferences were taken using a flexible tape measure, and waist-to-hip ratio (WHR) and waist-to-height ratio (WHtR) were calculated [23, 24]. Body composition components were measured using bioelectrical impedance analysis (BIA) with a Tanita BC-418 body composition analyzer. Measurements were performed in the morning, after an overnight fast of at least 8 h. Participants were asked to void their bladder immediately before the assessment and to avoid caffeine, alcohol, diuretics, large meals and physical activity for at least 12 h prior to measurement [25]. Fat mass index (FMI) and fat-free mass index (FFMI) were calculated using the following formulas [26]:

$$\mathrm{FMI}=\left(\mathrm{weight}\times\%\:\text{body fat}\right)/\mathrm{height}^2$$

$$ \mathrm{FFMI}=\left[\mathrm{weight}-\left(\mathrm{weight}\times\%\:\text{body fat}\right)\right]/\mathrm{height}^2$$

### Physical activity

Physical activity levels were assessed using the validated short-form International Physical Activity Questionnaire (IPAQ) [27]. This questionnaire measures low, moderate, and high levels of activities over the preceding seven days, expressed as MET-minutes per week. Based on these MET scores, participants were categorized into low (< 600 MET-minutes/week), moderate (600–3000 MET-minutes/week), and high (> 3000 MET-minutes/week) physical activity levels [28]. 

### Chronotype

Chronotype was determined using the Morningness-Eveningness Questionnaire (MEQ) by Horne and Ostberg. This self-reported instrument categorises individuals based on their preferred waking and sleeping hours, classifying them as morning, intermediate, or evening types [29, 30].

### Sleep quality

The quality of sleep over the past month was evaluated using the Pittsburgh Sleep Quality Index (PSQI). Each of the seven component scales within the PSQI has a score ranging from 0 to 3. A higher total score on the PSQI, which can range from 0 to 21, indicates poorer sleep quality. To differentiate between good and poor sleepers, a total PSQI score of more than 5 has demonstrated strong sensitivity and specificity (89.6% and 86.5%, respectively) [31]. 

### Dietary intake assessment

Participants’ dietary intake was assessed through three 24-hour dietary recalls, which enabled detailed collection of meal preparation methods and timing of food consumption [32]. The first recall was conducted in person by a trained interviewer, while the remaining two were completed on non-consecutive days via phone calls. For each recall, we recorded the type and quantity of foods consumed at each main meal, alongside meal-specific energy intake (kcal) and dietary components (g) using the Nutritionist IV software, based on the USDA National Nutrient Database [33]. Meals were categorized as breakfast, lunch, dinner, or snacks. When two or more meals were reported within a 59-minute interval, they were combined and averaged for serving time. Additionally, meals were classified as either main meals or snacks based on their contribution to total daily energy intake [34–36].

### Meal definitions

Eating events were identified from the 24‑hour dietary recalls and defined as any intake providing ≥ 50 kcal occurring at least 15 min apart from other events [35]. Participants reported the exact time of each event. Main meals were defined using standardized time windows, with the largest eating event between 05:00–11:00 classified as breakfast, 12:00–16:00 as lunch, and 17:00–23:00 as dinner. All other events were categorized as snacks. The mean number of eating events across the three recall days was calculated for each participant.

### Macronutrient quality assessment

In the present study, macronutrient quality was also categorized based on associated food groups into six subgroups: high-quality carbohydrates (whole grains, legumes, whole fruits, tomatoes, dark green vegetables, and other red/orange vegetables); low-quality carbohydrates (refined grains, fruit juice, potatoes, other starchy vegetables, and added sugars); animal protein (unprocessed red meat, processed meat, poultry, seafood, dairy products, and eggs); plant protein (whole grains, refined grains, legumes, nuts, and soy); saturated fatty acids (SFA); and unsaturated fatty acids (USFA). Dietary fat sources were not examined in detail due to their similarity to protein sources, as existing evidence on fats primarily focuses on fatty acid types rather than food sources [37]. Finally, after calculating the indices and determining macronutrient quantities from food groups, for each macronutrient quality index (high‑quality carbohydrates, saturated fat, unsaturated fat, animal protein, and plant protein), meal‑specific ratios were first calculated separately for breakfast and dinner. For example, the ratio for high‑quality carbohydrates at breakfast was defined as:

“High‑quality carbohydrate intake at breakfast ÷ total daily high‑quality carbohydrate intake,” and similarly for dinner.

The difference between dinner and breakfast ratios (Δ‑ratio) was then computed as:

$$\begin{aligned}&\triangle-\mathrm{ratio}\left(\text{dinner to breakfast}\right)\\&=\mathrm{Ratio}\_\mathrm{dinner}-\mathrm{Ratio}\_\mathrm{breakfast}.\end{aligned}$$

These Δ‑ratio values were subsequently categorized into tertiles for the main analyses. Tertiles of the Δ-ratio were created using the distribution of the overall study sample. Δ-ratio values were based on crude (non–energy-adjusted) data, and tertiles were not sex-specific. These tertile categories were used in all analyses.

### Statistical analysis

Statistical analyses were conducted using SPSS software version 27 and STATA 17 software. A p-value of less than 0.05 was regarded as statistically significant. The normality of data distribution was assessed using the Kolmogorov-Smirnov test. Demographic data, anthropometric measurements, and body composition indices were analyzed by gender using independent t-tests for quantitative variables and chi-square tests for qualitative variables. Quantitative data were reported as mean ± standard deviation, while qualitative data were presented as frequencies (percentages). Participants’ anthropometric measurements and body composition indices across macronutrient quality tertiles in dinner versus breakfast were calculated using ANCOVA. To examine the relationship between the differences in quality ratios of macronutrients consumed at dinner compared to breakfast with anthropometric measurements and body composition indices including BMI, waist-to-hip ratio, body fat percentage, lean body mass, and muscle mass, dose-response analysis using restricted cubic splines was performed. In the nonlinear analysis, confounders such as age, gender, education level, occupation, marital status, living situation, smoking status, menopause status in women, physical activity, BMI, time-oriented personality type, sleep quality, chronic disease status, supplement intake, and total energy intake were controlled. BMI was entered as an adjustment covariate in all models except when BMI was the outcome, to control for overall adiposity as a potential confounder. Multicollinearity diagnostics showed no concerning overlap (VIF < 3). To assess potential non-linear associations, restricted cubic spline models were fitted using cubic splines with three knots generated by the mkspline command (nk [3]; displayknots). The knot locations were obtained from the model output and used in subsequent spline-based analyses.

## Results

### Demographic characteristics of study participants

Among the participants from health centers, aged 20 to 59 years, 450 participants who had completed all questionnaires were included in the study. After excluding cases with under- or over-reporting of energy intake, 444 individuals were included in the statistical analyses. The absolute and relative frequency distribution of demographic characteristics including education level, occupation, marital status, living situation, physical activity level, smoking status, supplement use, chronic disease prevalence, and BMI were displayed in Table 1. Approximately 30% of participants had a desirable body mass index (BMI between 18.5 and 24.9), 1.6% were underweight (BMI less than 18.5), 45.3% were overweight (BMI between 25 and 29.9), and 23.4% were obese (BMI over 30). In both groups, the majority of individuals had university education (79.4% of men and 82.5% of women), were employed (88.2% of men and 63.8% of women), married (75% of men and 52.1% of women), non-smokers (88.2% of men and 96.7% of women), and healthy (73.5% of men and 81.7% of women). Significant differences were observed between men and women in terms of occupation (*P* < 0.001), marital status (*P* < 0.001), smoking status (*P* < 0.001), supplement use (*P* < 0.001), chronic disease prevalence (*P* = 0.039), and physical activity level (*P* = 0.03). Underweight and overweight statuses were more prevalent among men, while desirable BMI and obesity were more prevalent among women (*P* < 0.001).

**Table 1:** Demographic characteristics of study participants stratified by gender

Demographic characteristics	Total (444)	Men (204)	Women (240)
Age (mean± standard deviation)	38.24 ± 9.67	39.43 ± 9.45	37.22 ± 9.75
Education level			
Illiterate	9 (2%)	1 (0.5%)	8 (3.3%)
Below Diploma	18 (4.1%)	5 (2.5%)	13 (5.4%)
Diploma	57 (12.8%)	36 (17.6%)	21 (8.8%)
University	360 (81.1%)	162 (79.4%)	198 (82.5%)
Occupation			
Employed	333 (75%)	180 (88.2%)	153 (63.8%)
Housewife	63 (14.2%)	3 (1.5%)	60 (25%)
Retired	9 (2%)	3 (1.5%)	6 (2.5%)
Unemployed	39 (8.8%)	18 (8.8%)	21 (8.8%)
Marital status			
Single	144 (32.4%)	49 (24%)	95 (39.6%)
Married	278 (62.6%)	153 (75%)	125 (52.1%)
Divorced	10 (2.3%)	1 (0.5%)	9 (3.8%)
Widowed	12 (2.7%)	1 (0.5%)	11 (4.6%)
Physical activity level			
Low	204 (45.9%)	80 (39.2%)	124 (51.7%)
Moderate	151 (34%)	79 (38.7%)	72 (30%)
High	89 (20%)	45 (22.1%)	44 (18.3%)
Smoking status			
Non-smoker	396 (89.2%)	167 (81.9%)	229 (95.4%)
Ex-smoker	16 (3.6%)	13 (6.4%)	3 (1.3%)
Smoker	32 (7.2%)	24 (11.8%)	8 (3.3%)
Supplement use			
Yes	126 (28.4%)	29 (14.2%)	97 (40.4%)
No	318 (71.6%)	175 (85.8%)	143 (59.6%)
Living situation			
With someone	36 (8.1%)	21 (7.4%)	21 (8.8%)
Alone	408 (91.9%)	189 (92.6%)	219 (91.3%)
Chronic diseases			
Yes	98 (22.1%)	54 (26.5%)	44 (18.3%)
No	346 (77.9%)	150 (73.5%)	196 (81.7%)
BMI			
Underweight (< 18.5)	7 (1.6%)	6 (2.9%)	1 (0.4%)
Normal (18.5–24.9)	132 (29.7%)	42 (20.6%)	90 (37.5%)
Overweight (25–29.9)	201 (45.3%)	111 (54.4%)	90 (37.5%)
Obese (≥ 30)	104 (23.4%)	45 (22.1%)	24.6%)

Values are reported as mean and standard deviation or percentages. One-way ANOVA and Chi-square tests were used to evaluate categorical variables. Reported age values are presented as mean ± standard deviation, and comparisons of continuous variables were performed using the independent samples t-test

*Abbreviations*: *BMI* Body mass index

*P*-value < 0.05 is considered statistically significant

### Anthropometric measurements and body composition indices across macronutrient quality

Adjusted means of anthropometric indices and body composition across tertiles of macronutrient quality (carbohydrates, fats, and proteins) in dinner versus breakfast were reported in Table 2, respectively. According to the results of this analysis, no significant differences were observed in the anthropometric indices and body composition among the different tertiles of macronutrient quality in dinner compared to breakfast.

**Table 2:** Anthropometric measurements and body composition indices for different ratios of macronutrient quality at dinner compared to breakfast in the studied population

Difference in macronutrient quality ratio (Δ ratio)	Weight (kg)	Height (cm)	Waist circumference (cm)	Neck circumference (cm)	Waist-to-height ratio		Waist- to-hip ratio	Fat Percentage (%)	fat mass (kg)	Muscle mass (kg)		Fat free Mass (kg)	Fat mass Index (kg/m²)	Fat free Mass Index (kg/m²)
High-quality carbohydrates														
T_1_	65.97 ± 12.27	160.34 ± 5.74	87.95 ± 15.96	34.02 ± 2.59	0.55 ± 0.11		0.85 ± 0.15	32.75 ± 8.22	22.73 ± 8.89	41.00 ± 3.90		43.24 ± 4.19	8.71 ± 3.30	18.66 ± 2.58
T_2_	69.05 ± 13.04	160.32 ± 8.19	86.06 ± 11.81	34.27 ± 2.72	0.54 ± 0.08		0.82 ± 0.72	34.35 ± 7.04	24.59 ± 8.88	42.54 ± 4.99		44.82 ± 5.26	8.60 ± 3.53	18.51 ± 3.11
T_3_	70.37 ± 12.92	161.10 ± 6.79	89.73 ± 14.07	34.71 ± 2.56	0.56 ± 0.09		0.84 ± 0.10	35.33 ± 6.53	25.59 ± 8.77	42.54 ± 4.68		44.84 ± 4.95	8.21 ± 3.54	18.46 ± 3.09
* P-value*	0.92	0.96	0.14	0.66	0.10		0.16	0.81	0.97	0.72		0.70	0.49	0.87
Low-quality carbohydrates														
T_1_	68.38 ± 12.83	160.41 ± 7.03	86.21 ± 13.41	34.20 ± 2.81	0.54 ± 0.09		0.82 ± 0.08	33.78 ± 7.55	24.10 ± 8.82	42.02 ± 4.54		44.31 ± 4.78	8.41 ± 3.80	18.39 ± 3.39
T_2_	68.81 ± 11.23	160.27 ± 5.55	89.57 ± 13.46	34.64 ± 2.40	0.56 ± 0.09		0.85 ± 0.10	34.91 ± 6.67	24.76 ± 7.99	42.14 ± 4.40		44.44 ± 4.68	8.60 ± 3.08	18.43 ± 2.53
T_3_	68.46 ± 14.52	159.96 ± 8.45	88.07 ± 15.04	34.21 ± 2.69	0.55 ± 0.10		0.84 ± 0.14	33.97 ± 7.64	24.25 ± 9.93	41.96 ± 4.94		44.26 ± 5.26	8.60 ± 3.35	18.71 ± 2.64
* P-value*	0.85	0.91	0.10	0.16	0.09		0.09	0.20	0.50	0.90		0.92	0.96	0.84
SFA														
T_1_	68.96 ± 12.07	160.04 ± 7.39	87.98 ± 12.99	34.57 ± 2.69	0.55 ± 0.09		0.83 ± 0.07	34.36 ± 7.24	24.61 ± 8.50	42.55 ± 4.73		44.90 ± 4.96	8.69 ± 3.59	18.80 ± 3.29
T_2_	67.66 ± 12.69	160.68 ± 5.76	86.96 ± 13.95	34.20 ± 2.43	0.54 ± 0.09		0.83 ± 0.10	33.92 ± 7.25	23.74 ± 9.04	41.64 ± 4.20		43.91 ± 4.46	8.34 ± 3.19	18.04 ± 2.45
T_3_	69.64 ± 13.73	160.00 ± 7.95	88.94 ± 14.74	34.34 ± 2.79	0.56 ± 0.10		0.84 ± 0.14	34.55 ± 7.43	25.05 ± 9.11	42.12 ± 4.93		44.41 ± 5.25	8.45 ± 3.66	18.87 ± 3.07
* P-value*	0.77	0.88	0.79	0.69	0.83		0.81	0.56	0.68	0.45		0.39	0.51	0.30
USFA														
T_1_	69.24 ± 13.40	160.91 ± 7.24	87.91 ± 12.85	34.54 ± 2.44	0.55 ± 0.09		0.83 ± 0.07	34.23 ± 7.27	24.53 ± 9.33	42.39 ± 4.64		44.69 ± 4.93	8.40 ± 3.49	18.72 ± 3.14
T_2_	68.05 ± 11.82	160.10 ± 7.72	86.84 ± 11.95	34.17 ± 2.71	0.54 ± 0.08		0.83 ± 0.07	34.12 ± 7.36	24.15 ± 8.25	41.61 ± 3.99		43.89 ± 4.27	8.41 ± 3.55	18.48 ± 2.80
T_3_	68.74 ± 13.53	159.69 ± 5.75	89.33 ± 17.15	34.38 ± 2.76	0.56 ± 0.11		0.84 ± 0.17	34.49 ± 7.31	24.71 ± 9.22	42.29 ± 5.27		44.62 ± 5.54	8.67 ± 3.35	18.42 ± 2.90
* P-value*	0.34	0.23	0.68	0.32	0.66		0.87	0.67	0.71	0.24		0.26	0.97	0.57
Animal proteins														
T_1_	68.18 ± 12.48	159.91 ± 9.28	85.98 ± 12.93	34.14 ± 2.43	0.54 ± 0.10		0.81 ± 0.07	33.86 ± 7.73	23.92 ± 9.11	41.96 ± 4.08		44.23 ± 4.34	8.21 ± 3.80	18.38 ± 3.27
T_2_	68.21 ± 11.99	160.50 ± 6.15	88.05 ± 13.69	34.74 ± 2.56	0.55 ± 0.08		0.83 ± 0.10	34.59 ± 5.85	24.19 ± 7.83	41.81 ± 4.58		44.08 ± 4.84	8.66 ± 2.99	18.55 ± 2.46
T_3_	69.38 ± 14.01	160.33	89.48 ± 14.93	34.18 ± 2.85	0.56 ± 0.10		0.84 ± 0.14	34.23 ± 8.06	25.04 ± 9.62	42.42 ± 5.13		44.77 ± 5.43	8.62 ± 3.55	18.67 ± 3.07
* P-value*	0.46	0.91	0.04	0.02	0.08		0.08	0.07	0.17	0.89		0.85	0.71	0.90
Plant proteins														
T_1_	69.47 ± 12.10	160.37 ± 7.11	88.66 ± 13.73	34.64 ± 2.45	0.55 ± 0.10		0.83 ± 0.08	34.42 ± 7.78	24.91 ± 8.65	42.23 ± 4.17		44.55 ± 4.39	8.15 ± 3.37	18.76 ± 3.35
T_2_	69.77 ± 14.20	160.50 ± 5.96	89.56 ± 15.27	34.63 ± 3.02	0.56 ± 0.10		0.85 ± 0.10	34.86 ± 7.62	25.28 ± 10.03	42.22 ± 4.65		44.54 ± 4.94	8.87 ± 3.35	18.32 ± 2.74
T_3_	66.70 ± 12.07	159.90 ± 7.94	85.55 ± 12.54	33.81 ± 2.32	0.54 ± 0.09		0.82 ± 0.13	33.44 ± 6.42	23.07 ± 7.81	41.78 ± 5.02		44.04 ± 5.32	8.46 ± 3.65	18.54 ± 2.77
* P-value*	0.10	0.10	0.20	0.11	0.52		0.32	0.29	0.06	0.85		0.81	0.46	0.74

Values are reported as mean ± standard deviation. P values were obtained from analysis of covariance, with significance set at *P* < 0.05. *P* values were adjusted for variables including age, gender, educational level, occupation, marital status, living conditions, smoking status, menopausal status in women, physical activity, body mass index, time-oriented personality type, sleep quality, chronic diseases, supplement intake, and and the quality of macronutrients at lunch

### Daily macronutrient quantity across macronutrient quality ratios

Table 3 presents the total daily intake of energy and absolute macronutrients (carbohydrates, proteins, and fats) across tertiles of the dinner‑to‑breakfast macronutrient quality ratios. Overall, daily intakes of carbohydrates and fats did not differ significantly across tertiles of any of the examined macronutrient quality ratios (all *P* > 0.05). However, significant differences were observed in total daily protein intake across tertiles of the animal protein ratio (*P* = 0.04) and in daily energy intake across tertiles of the plant protein ratio (*P* = 0.01).

**Table 3:** Daily macronutrient quantity across ratios of macronutrient quality at dinner compared to breakfast in the studied population

Difference in macronutrient quality ratio (Δ ratio)	Energy (kcal/day)	Carbohydrates (gr/day)	Proteins (gr/day)	Fats (gr/day)
High-quality carbohydrates				
T_1_	1917 ± 597	266.79 ± 90.1	73.38 ± 27.48	67.2 ± 27.13
T_2_	1963 ± 621	271.88 ± 95.2	74.18 ± 27.24	73.11 ± 48.21
T_3_	1862 ± 492	248.79 ± 81.65	71.64 ± 24.11	68.98 ± 24.18
* P-value*	0.24	0.15	0.96	0.41
Low-quality carbohydrates				
T_1_	1928 ± 710	260.92 ± 85.91	76.06 ± 27.87	73.86 ± 47.05
T_2_	1947 ± 665	269 ± 50±101.06	72.80 ± 27.47	68.94 ± 28.14
T_3_	1867 ± 527	257.17 ± 80.29	70.65 ± 23.03	66.28 ± 25.33
* P-value*	0.60	0.40	0.10	0.21
SFA				
T_1_	1854 ± 589	260.93 ± 90.12	69.86 ± 25.61	63.84 ± 26.16
T_2_	1959 ± 566	267.32 ± 85.99	76.31 ± 29.4	73.96 ± 47.41
T_3_	1922 ± 567	258.67 ± 92.57	72.92 ± 23.37	71.03 ± 26.38
* P-value*	0.32	0.74	0.43	0.2
USFA				
T_1_	1936 ± 574	268.21 ± 87.36	75.39 ± 29.08	71.68 ± 47.61
T_2_	1957 ± 568	264.97 ± 85.56	75.07 ± 25.42	71.76 ± 26.58
T_3_	1844 ± 579	253.74 ± 95.18	68.67 ± 23.74	65.47 ± 25.93
* P-value*	0.17	0.53	0.33	0.6
Animal proteins				
T_1_	1944 ± 619	270 ± 98.85	71.36 ± 26.81	70.03 ± 28.71
T_2_	1886 ± 528	256.84 ± 79.26	74.08 ± 28.2	71.17 ± 46.19
T_3_	1914 ± 572	260.88 ± 89.37	73.85 ± 23.73	67.97 ± 26.5
* P-value*	0.69	0.46	0.04	0.47
Plant proteins				
T_1_	1925 ± 534	263.77 ± 85.55	73.41 ± 24.29	68.98 ± 26.61
T_2_	2002 ± 629	276.25 ± 95.45	77.73 ± 29.57	74.09 ± 48.45
T_3_	1814 ± 538	247.21 ± 84.98	68.05 ± 23.74	65.97 ± 23.44
* P-value*	0.01	0.88	0.26	0.76

Values are reported as mean ± standard deviation. *P* values were obtained from analysis of covariance, with significance set at *P* < 0.05. Values were adjusted for variables including age, physical activity, body mass index, and energy intake, except for energy intake itself

*SFA* Saturated fatty acids, *USFA* Unsaturated fatty acids

Despite these isolated differences, most categories showed consistent absolute daily macronutrient intakes across tertiles. This suggests that the observed associations in our study are primarily driven by the quality and timing of macronutrient intake rather than their total daily quantity.

### Non-linear dose-response associations

**Fig. 1:**
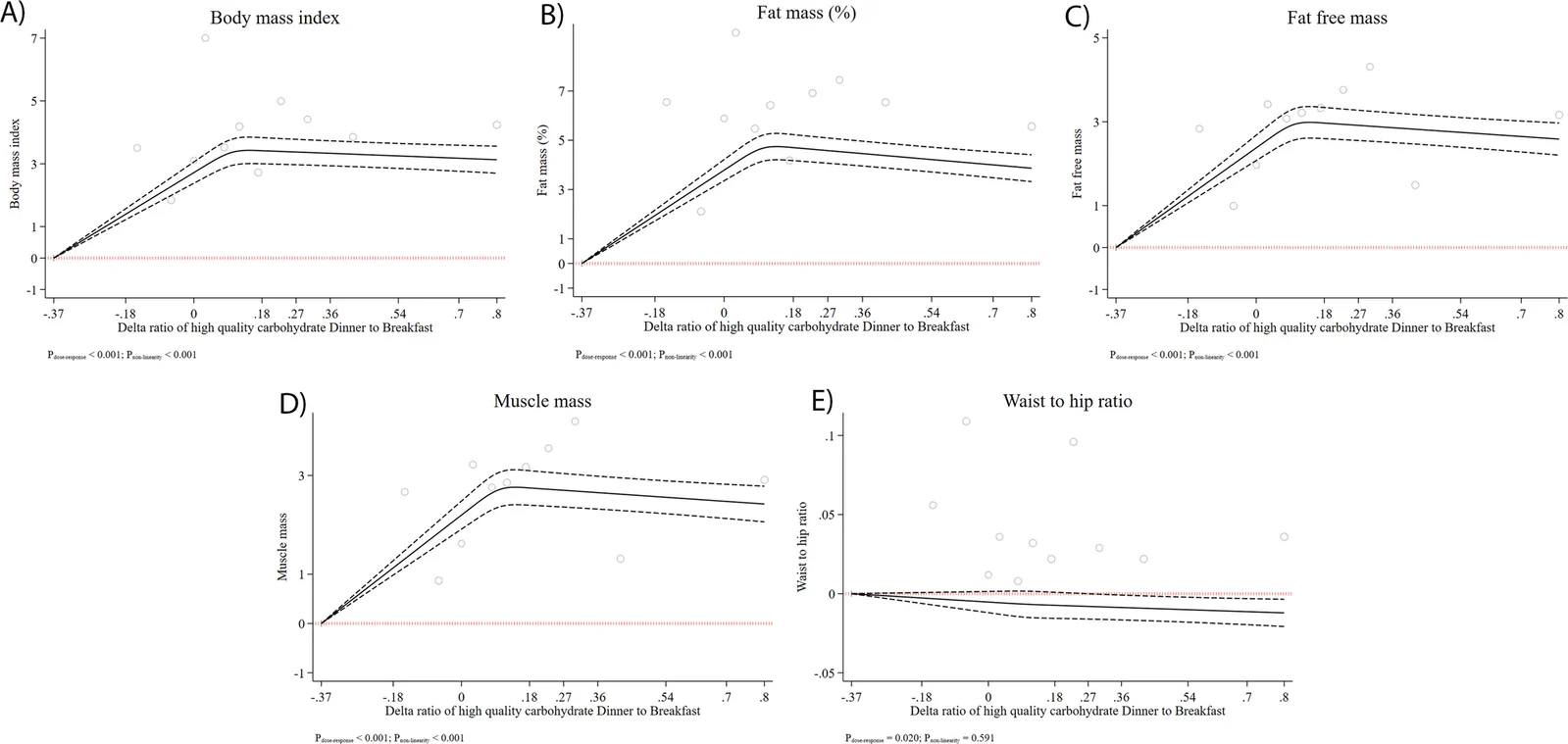
Dose-response relationship between the difference in the ratio of high-quality carbohydrates at dinner compared to breakfast and body mass index **A**, body fat percentage **B**, fat-free mass **C**, muscle mass **D**, and waist-to-hip ratio **E**

**Fig. 2:**
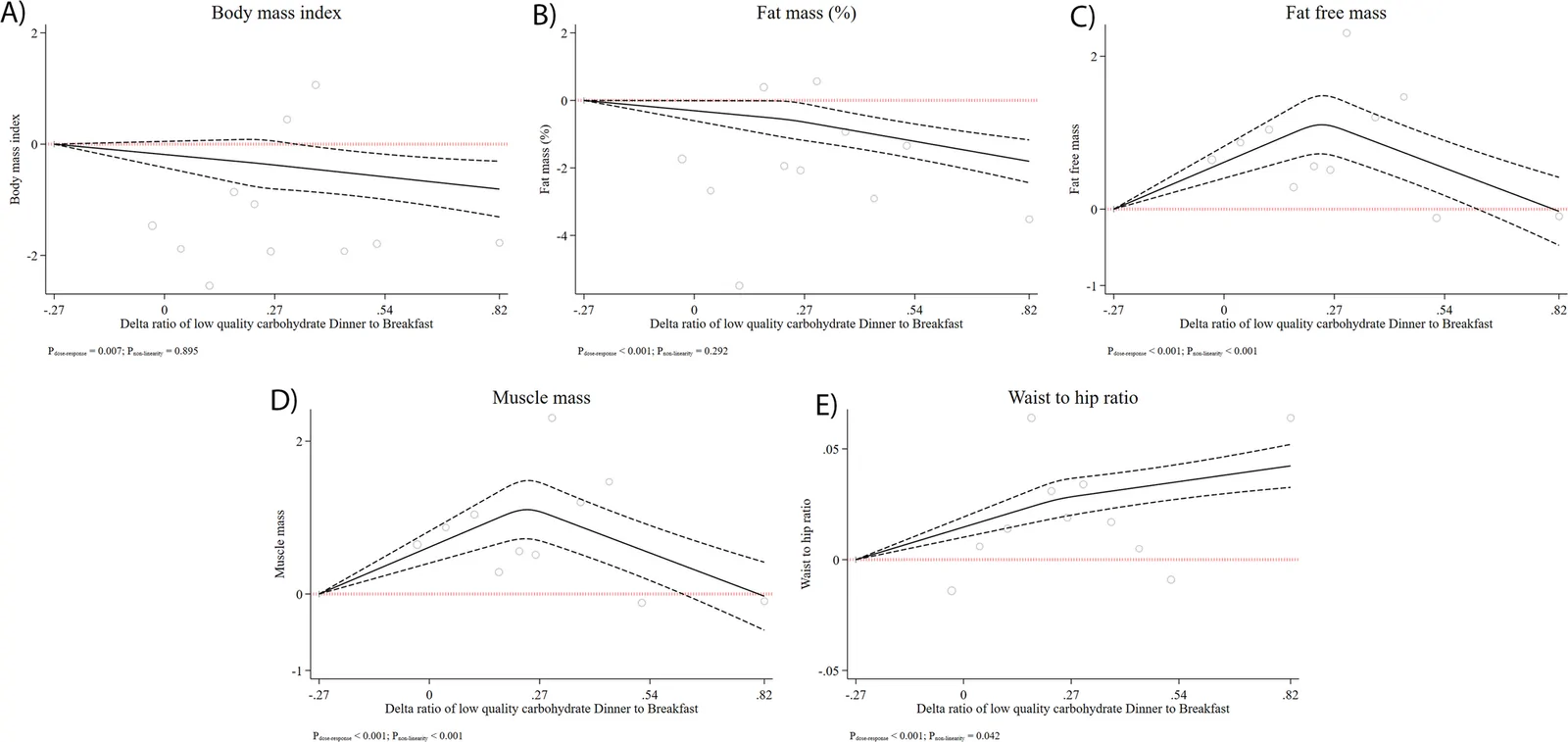
Dose-response relationship between the difference in the ratio of saturated fats at dinner compared to breakfast and body mass index **A**, body fat percentage **B**, fat-free mass **C**, muscle mass **D**, and waist-to-hip ratio **E**

**Fig. 3:**
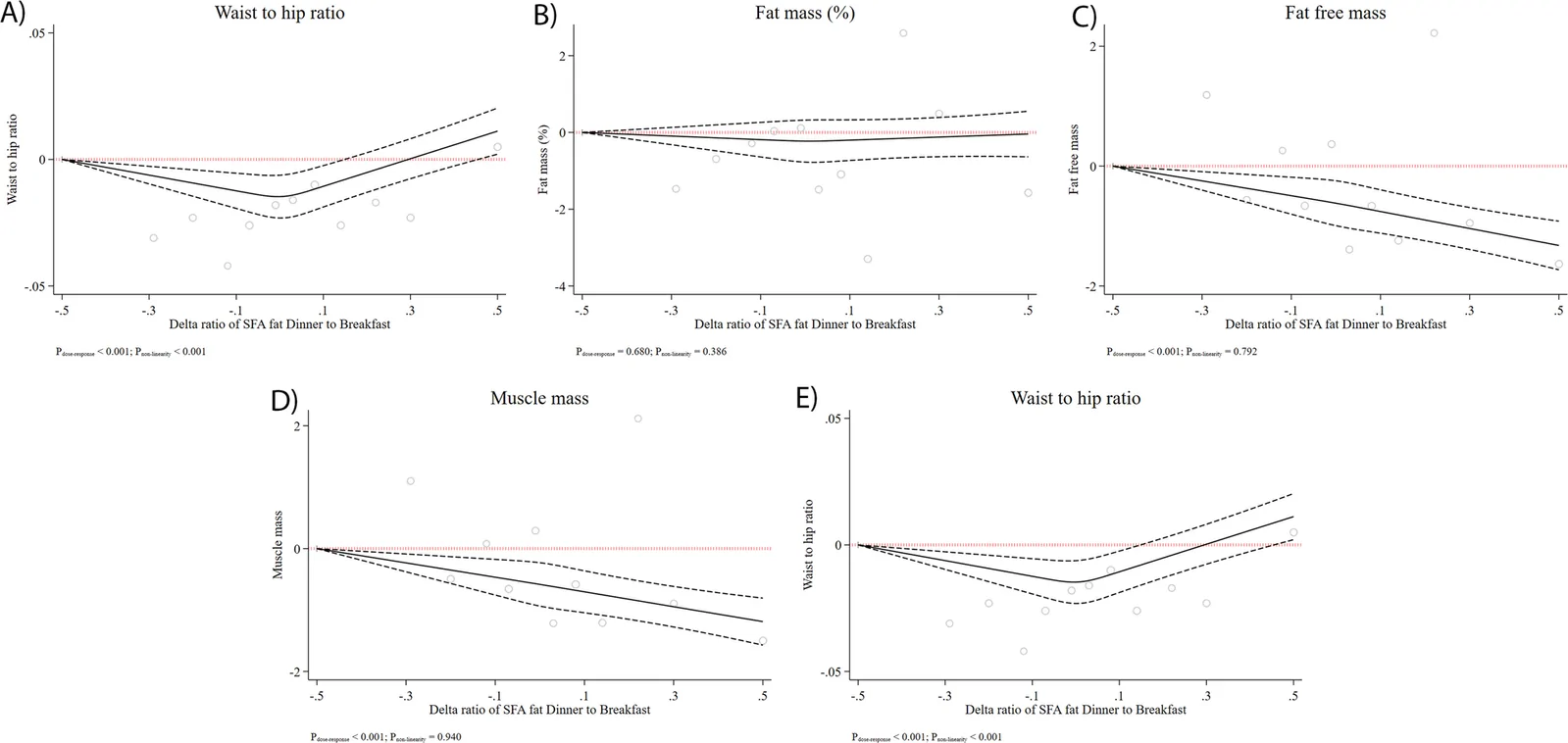
Dose-response relationship between the difference in the ratio of low-quality carbohydrates at dinner compared to breakfast and body mass index **A**, body fat percentage **B**, fat-free mass **C**, muscle mass **D**, and waist-to-hip ratio **E**

**Fig. 4:**
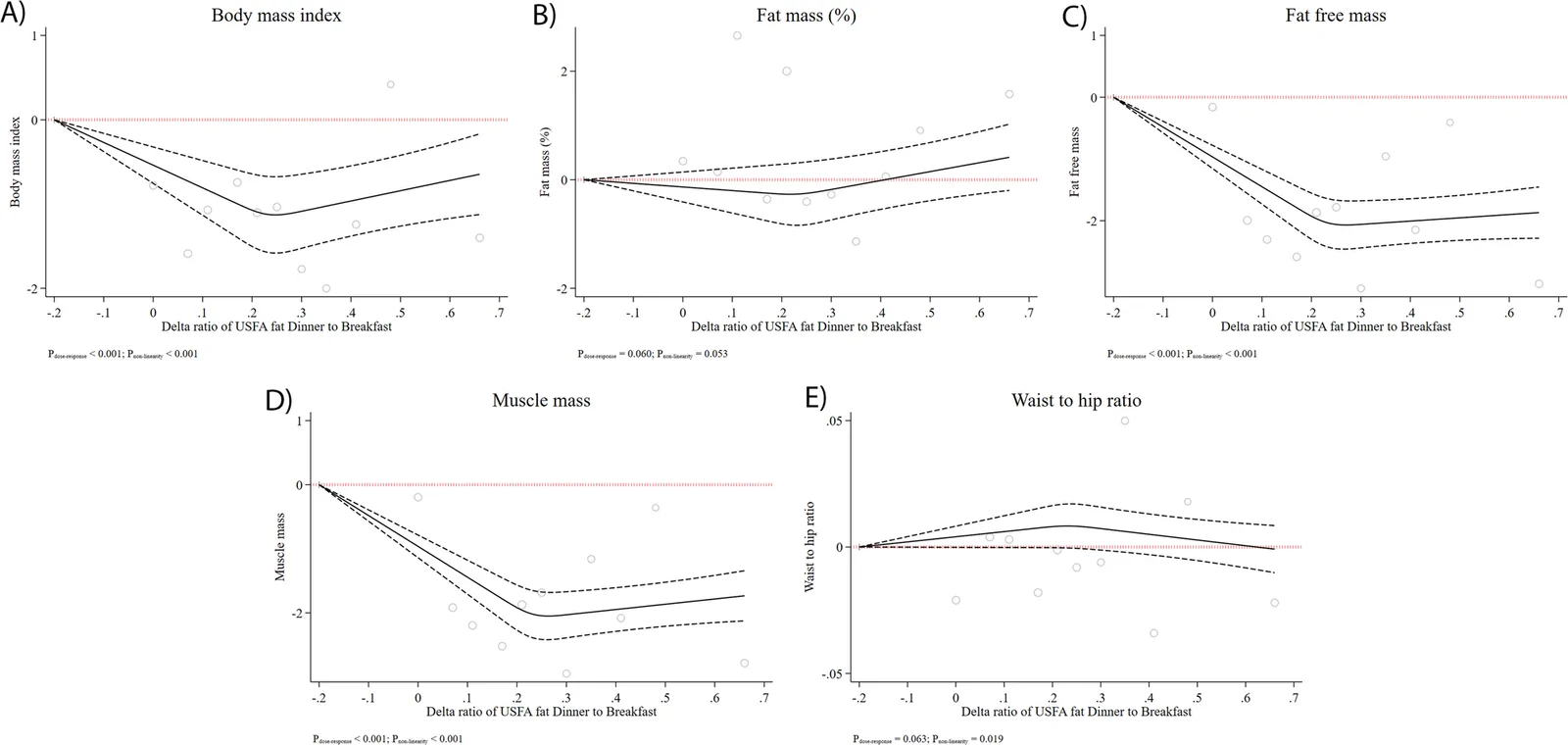
Dose-response relationship between the difference in the ratio of unsaturated fats at dinner compared to breakfast and body mass index **A**, body fat percentage **B**, fat-free mass **C**, muscle mass **D**, and waist-to-hip ratio **E**

**Fig. 5:**
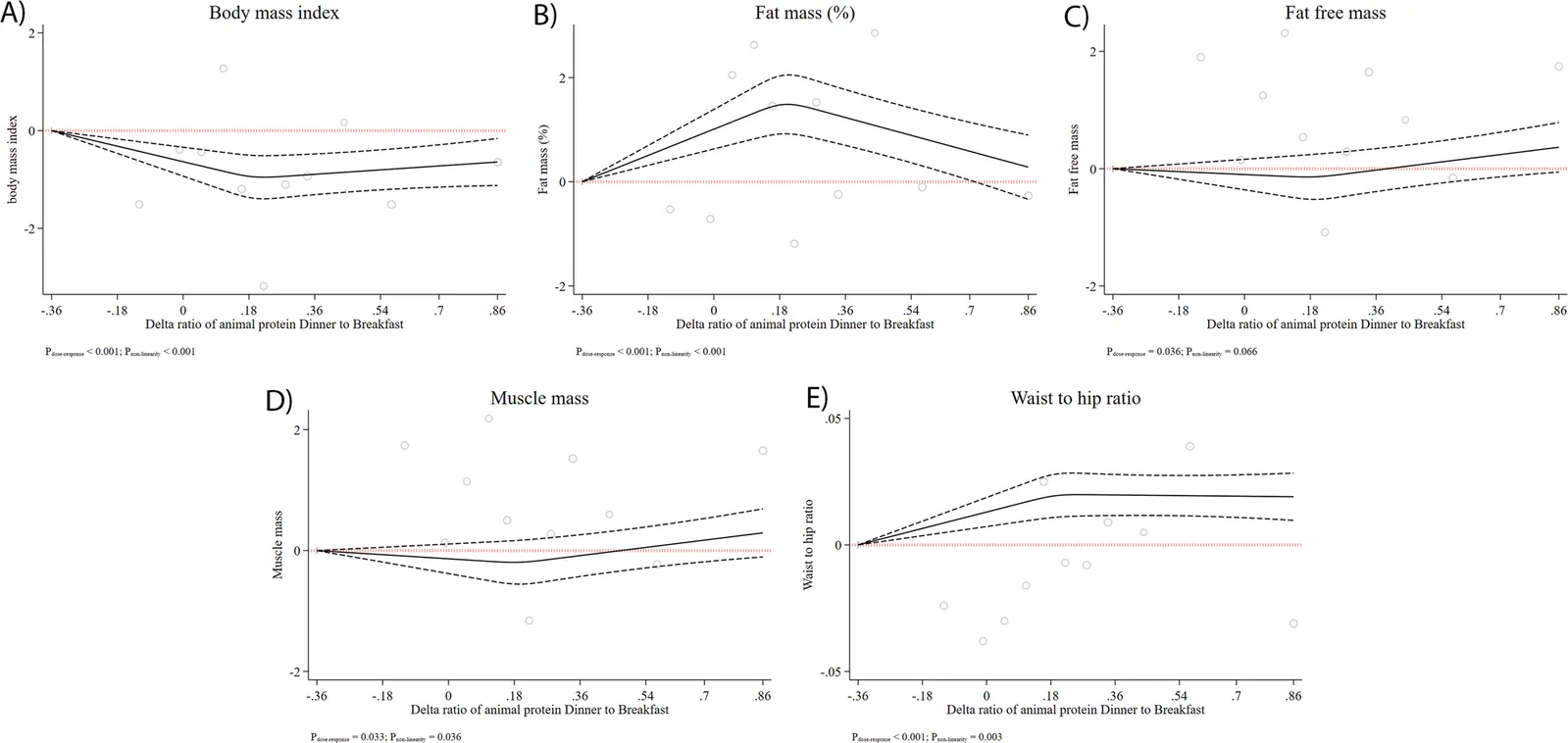
Dose-response relationship between the difference in the ratio of animal proteins at dinner compared to breakfast and body mass index **A**, body fat percentage **B**, fat-free mass **C**, muscle mass **D**, and waist-to-hip ratio **E**

**Fig. 6:**
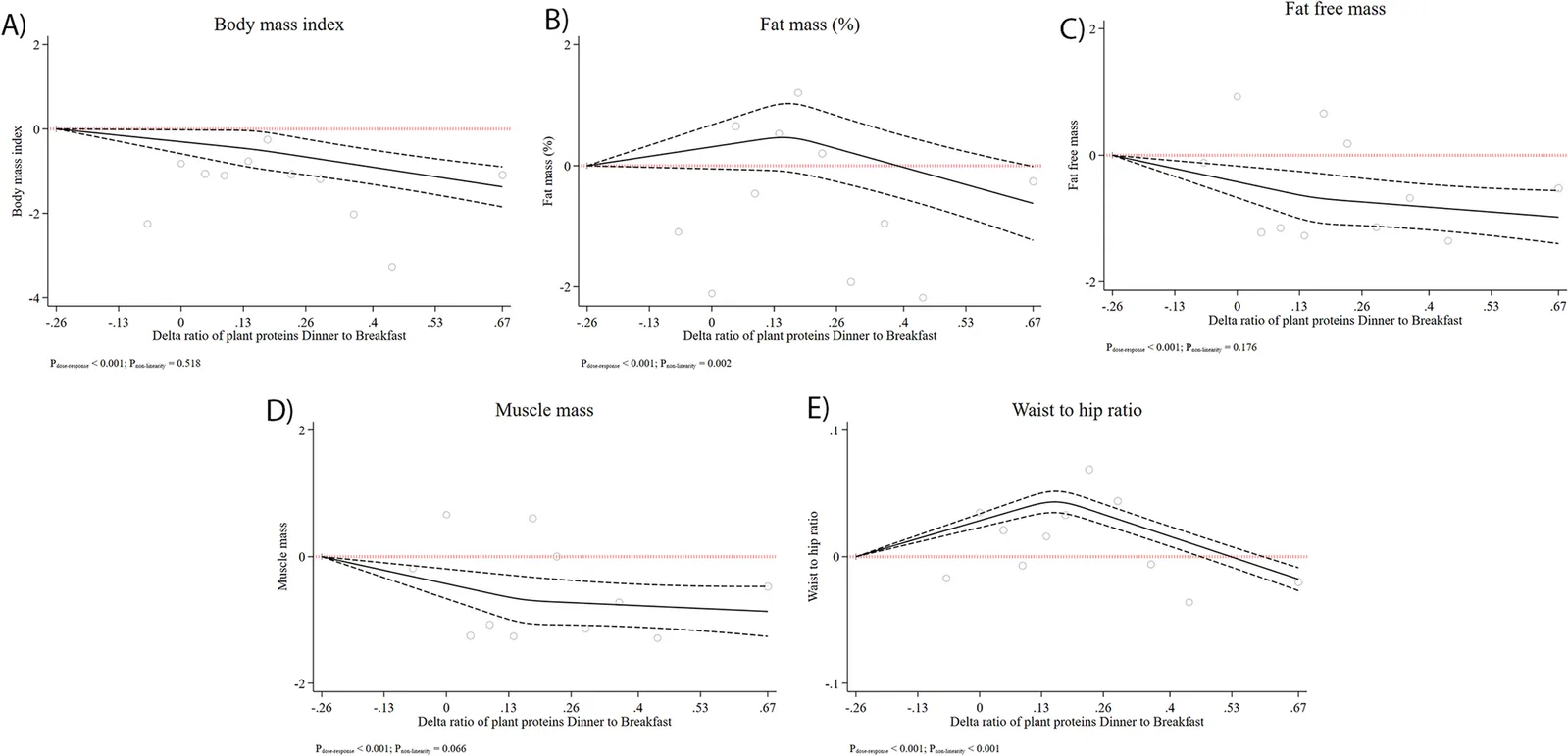
Dose-response relationship between the difference in the ratio of plant proteins at dinner compared to breakfast and body mass index **A**, body fat percentage **B**, fat-free mass **C**, muscle mass **D**, and waist-to-hip ratio **E**

Restricted cubic spline analyses were performed to examine the dose-response associations between the difference in macronutrient quality ratio at dinner compared with breakfast and body composition indices (Figs. 1, 2, 3, 4, 5 and 6).

Figure 1 examined the dose-response associations between the difference in the ratio of high-quality carbohydrates at dinner compared to breakfast and body composition indices. inverted U-shaped associations were observed for BMI (Fig. 1A), body fat percentage (Fig. 1B), fat-free mass (Fig. 1C) and muscle mass (Fig. 1D) (all P_non−linearity_ <0.001). While Fig. 1E, showed that with an increase in the difference in the high-quality carbohydrate ratio, the waist-to-hip ratio significantly decreased linearly (P_dose−response_=0.02; P_non−linearity_=0.59).

For low-quality carbohydrates in Fig. 2, BMI (Fig. 2A) (P_dose−response_=0.007; P_non−linearity_ =0.89) and body fat percentage (Fig. 2B) (P_dose−response_ <0.001; P_non−linearity_ =0.29) showed significant linear decreases with increasing dinner-to-breakfast ratio, whereas fat-free mass (Fig. 2C) and muscle mass (Fig. 2D) demonstrated inverted U-shaped associations (P_dose−response_ <0.001; P_non−linearity_<0.001). For Fig. 2E, the results indicated that as the difference in the ratio of low-quality carbohydrates increased, the waist-to-hip ratio rose significantly (P_dose−response_ <0.001; P_non−linearity_ =0.04).

In Fig. 3 for saturated fat, significant U-shaped associations were observed for BMI (Fig. 3A) and waist-to-hip ratio (Fig. 3E) (P_dose−response and non−linearity_ <0.001), while fat-free mass (Fig. 3C) (P_dose−response_ <0.001; P_non−linearity_ =0.79) and muscle mass (Fig. 3D) (P_dose−response_ <0.001; P_non−linearity_ =0.94) showed significant linear decreases as the dinner-to-breakfast ratio increased. No significant association was found for body fat percentage (Fig. 3B) (P_dose−response_=0.68; P_non−linearity_ =0.39).

Figure 4 illustrated the dose-response relationship between the difference in the ratio of unsaturated fat intake at dinner compared to breakfast and various body composition indices. According to Fig. 4, BMI (Fig. 4A), fat-free mass (Fig. 4C), and muscle mass (Fig. 4D) demonstrated U-shaped associations (P_dose−response and non−linearity_ <0.001), whereas body fat percentage (Fig. 4B) showed no significant association (P_dose−response_ =0.06; P_non−linearity_ =0.053). Waist-to-hip ratio (Fig. 4E) showed a significant non-linear association despite the absence of a significant linear trend (P_dose−response_ =0.06; P_non−linearity_ =0.019).

Based on the results of Fig. 4 regarding animal proteins, BMI (Fig. 5A) showed a mild U-shaped association (P_dose−response and non−linearity_ <0.001) and body fat percentage (Fig. 5B) demonstrated an inverted U-shaped relationship (P_dose−response and non−linearity_ <0.001). Fat-free mass (Fig. 5C) (P_dose−response_=0.036; P _non−linearity_ =0.07) and muscle mass (Fig. 5D) (P_dose−response_=0.033; P _non−linearity_ =0.036) showed a slight reverse U-shaped association, while in Fig. 5E, waist-to-hip ratio increased linearly with higher animal protein intake at dinner compared with breakfast (P_dose−response_ <0.001).

For plant protein in Fig. 6, BMI (Fig. 6A) showed a significant linear decrease (P_dose−response_ <0.001), while body fat percentage (Fig. 6B) (P_dose−response_ <0.001; P _non−linearity_=0.002) and waist-to-hip ratio (Fig. 6E) (P_dose−response and non−linearity_ <0.001) demonstrated significant inverted U-shaped associations. In contrast, fat-free mass (Fig. 6C) (P_dose−response_<0.001; P _non−linearity_ =0.176) and muscle mass (Fig. 6D) (P_dose−response_<0.001; P _non−linearity_=0.066) showed significant monotonic declines with increasing plant protein intake at dinner versus breakfast.

## Discussion

In the present study, a greater intake of high-quality carbohydrates at dinner versus breakfast was associated with a more favorable waist-to-hip ratio, BMI, and body fat percentage. In contrast, higher saturated fat intake at dinner was associated with greater adiposity and lower fat-free and muscle mass. Unsaturated fats demonstrated a U-shaped association, with moderate intake linked to more favorable outcomes. Higher plant protein intake at dinner was associated with lower BMI and waist-to-hip ratio, whereas higher animal protein intake was related to greater lean mass but less favorable fat distribution.

### High-quality and low-quality carbohydrates

High-quality carbohydrates consumed at dinner compared to breakfast, showed a complex non-linear association with body composition, exhibiting threshold effects where BMI and body fat percentage initially increased but later stabilized or declined, while waist-to-hip ratio decreased linearly. Additionally, Higher ratios of low-quality carbohydrates at dinner was associated with lower BMI and body fat percentage linearly, but increased waist-to-hip ratio. Fat-free and muscle mass initially increased but declined after a peak. These findings align with studies emphasizing the metabolic benefits of high-quality carbohydrates when distributed across meals. Reynolds et al. (2019) conducted a meta-analysis demonstrating that diets rich in dietary fiber (a marker of high-quality carbohydrates) are associated with lower body weight [38]. Similarly, Jenkins et al. (2002) reported that low glycemic index carbohydrate sources improve post-meal glucose levels metabolism, supporting better weight regulation [39]. Another dose-response meta-analysis has shown that Overweight/obese females can shift their carbohydrate intake for higher cereal fiber to decrease T2DM risk, but GL may cancel out this effect [40]. The findings of a randomized controlled trial have revealed that the carbohydrate-rich meals consumed at dinner lead to worse postprandial glucose homeostasis compared to breakfast, independent of glycemic index [41], underscoring the significance of carbohydrate timing in optimizing body composition and reducing type 2 diabetes risk. Hou et al. found that consuming more high-quality carbohydrates at dinner was associated with lower all-cause and cardiovascular mortality, while higher low-quality carbohydrate intake at dinner increased diabetes mortality risk. Substituting low-quality with high-quality carbohydrates at dinner significantly reduced mortality risks, highlighting the importance of carbohydrate timing and quality for long-term health [42]. The findings of our study appear to contradict findings associating low-quality carbohydrate intake at dinner with increased diabetes mortality. This apparent discrepancy can be explained by recognizing that while higher low-quality carbohydrate intake at dinner may reduce BMI and body fat percentage in the short term, these benefits are offset by increased central fat deposition, poor postprandial glucose regulation, and long-term risks of insulin resistance and diabetes-related mortality. This underscores the importance of distinguishing between immediate body composition changes and long-term health outcomes when interpreting such results. Furthermore, the decline in fat-free and muscle mass after a peak further supports the idea that higher low-quality carbohydrate intake at dinner could lead to metabolic inefficiencies over time, potentially explaining the increased mortality risk in long-term studies. Results of the AWHS study suggested that there is a protective association between the consumption of high-quality carbohydrates and the presence of MetS and hypertriglyceridemia among middle-aged men free of CVD [43]. Mechanistically, diurnal variations in insulin sensitivity provide a plausible explanation for these results. Insulin sensitivity is highest in the morning, facilitating better glucose uptake and glycogen synthesis [44]. Consuming high-quality carbohydrates in the evening may lead to increased post-meal glucose levels and fat storage due to reduced metabolic efficiency. However, metabolic adaptations can attenuate these effects over time. Supporting this, a systematic review highlighted that evening carbohydrate intake above specific thresholds may disrupt circadian glucose homeostasis, exacerbating adiposity [45]. Additionally, another study noted that meal timing interacts with circadian rhythms to influence energy partitioning and fat storage, making the timing and quality of carbohydrates critical for optimizing body composition [46].

### Saturated and unsaturated fats

Higher saturated fat intake at dinner versus breakfast was associated with higher BMI and waist-to-hip ratio, while fat-free and muscle mass declined linearly. Furthermore, a U-shaped relationship between unsaturated fat intake and BMI, fat-free mass, and muscle mass was observed. These findings align with de Souza et al. (2015), who demonstrated in a meta-analysis that saturated fat consumption is associated with increased BMI and central adiposity [47]. The study of Frankenberg et al. has revealed that a diet very high in fat and saturated fat adversely affects insulin sensitivity and thereby might contribute to the development of type 2 diabetes, regardless of time-eating [48]. Another study provides mechanistic insights linking both saturated and unsaturated fats to circadian and metabolic regulation. Palmitate, a proinflammatory saturated fat, disrupts circadian rhythms and induces time-dependent inflammatory responses, impairing lipid metabolism and contributing to adiposity and muscle loss, particularly when consumed in the evening. In contrast, DHA, an anti-inflammatory unsaturated fat, mitigates these effects by repressing inflammatory signaling and stabilizing circadian timekeeping. These findings align with our study, where saturated fats increased BMI and waist-to-hip ratio, while moderate unsaturated fat intake improved body composition. They underscore the importance of replacing saturated fats with unsaturated alternatives, particularly at dinner, to optimize metabolic health and circadian function [49]. Another study highlights the significant metabolic benefits of replacing saturated fats with monounsaturated fats, resulting in reduced body weight and fat mass despite consistent energy intake. These findings parallel our observations, where saturated fat consumption increased BMI and central adiposity, while unsaturated fats supported improved body composition. The distinct advantages of unsaturated fats likely stem from their anti-inflammatory properties and enhanced lipid metabolism [50].

### Animal and plant proteins

Based on our results, higher animal protein intake at dinner was associated with higher BMI, fat-free mass, and muscle mass, but also elevated waist-to-hip ratio and body fat percentage. Moreover, plant protein intake at dinner was linked to reductions in BMI and waist-to-hip ratio but also decreased fat-free mass and muscle mass linearly. Several systematic reviews and meta-analyses support our findings that animal protein intake is more beneficial for lean mass development compared to plant protein, particularly in younger adults. The higher leucine content and complete amino acid profile of animal proteins likely explain their superior anabolic effect [51–54]. Another study supports our findings by emphasizing the role of protein timing and energy balance in optimizing body composition [55]. Another study found that protein intake at breakfast was inversely associated with systolic and diastolic blood pressure and positively associated with HDL-cholesterol levels, indicating potential cardiovascular benefits. In contrast, this suggested a higher risk of insulin resistance with evening protein consumption. Notably, protein intake at meals and snacks showed no significant associations with BMI and waist circumference. However, the study did not specify the source of protein (animal vs. plant) [56]. However, current evidence supports the idea that CVD risk can be reduced by a dietary pattern that provides more plant sources of protein compared with the typical American diet and also included animal-based protein foods that are unprocessed and low in saturated fat [57, 58]. The results about animal proteins can be justified by the interplay of circadian biology and the nutrient-specific properties of animal proteins. In the evening, reduced insulin sensitivity favors lipogenesis and fat storage. Additionally, elevated cortisol levels in the evening, potentially exacerbated by high animal protein intake [59], promote central fat deposition. Despite these effects, animal proteins stimulate muscle protein synthesis via leucine-mediated activation of the mTOR pathway [60], contributing to increased muscle mass and fat-free mass.

Regarding the results of plant proteins, the fiber content enhances satiety, and improves lipid metabolism [61], leading to lower overall caloric intake and reduced fat storage and BMI. However, the lower bioavailability of plant proteins, due to anti-nutritional factors and their incomplete amino acid profiles limit their efficacy in promoting muscle protein synthesis. reduced leucine content in plant proteins results in suboptimal activation of the mTOR pathway, further impairing muscle protein synthesis. In the evening, when metabolic rates and protein turnover are lower, these limitations are amplified, resulting in a decline in MM and FFM despite the beneficial effects on adiposity and body composition [52, 62].

### Chrono-nutrition

Our findings may also be interpreted within the framework of chrono-nutrition, which emphasizes the importance of the timing of nutrient intake in relation to the circadian rhythm. Glucose metabolism [63], insulin sensitivity [64], and diet-induced thermogenesis [65] exhibit clear diurnal variation, with metabolic efficiency generally being higher earlier in the day and declining toward the evening. Greater evening intake may lead to circadian misalignment [66, 67], impaired postprandial glucose handling [68], reduced lipid oxidation [69, 70], increased adiposity [71, 72] and adverse body composition [73], whereas earlier intake may better align with endogenous metabolic rhythms [74]. This concept of circadian alignment versus misalignment could partly explain why the associations observed in our study may be related to differences in diurnal metabolic responses rather than macronutrient quality or distribution alone.

### Strengths and limitations

This study demonstrates significant strengths, including the innovative evaluation of macronutrient quality ratios across main meals, addressing a gap in previous research. The rigorous adjustment for a broad spectrum of confounding variables enhances the validity and reliability of the findings. Moreover, the examination of dose-response relationships offers detailed insights into the non-linear effects of macronutrient distribution on body composition, providing nuanced insights into the non-linear effects of macronutrient distribution on body composition. However, the cross-sectional design of this study limits the ability to draw causal inferences, as it identifies correlations without establishing cause-and-effect relationships. Additionally, the reliance on 24-hour dietary recalls may introduce reporting bias, potentially affecting the accuracy of the dietary data. The study’s population, being regionally specific, further limits the generalizability of the findings to broader cultural or geographical contexts. Furthermore, despite adjustment for multiple potential confounders, residual confounding cannot be entirely excluded given the observational nature of the study.

## Conclusion

We found that greater consumption of high-quality carbohydrates, unsaturated fats, and plant-based proteins at dinner compared to breakfast may be associated with optimized body composition. Nevertheless, further longitudinal studies, particularly those using substitution models, are warranted to investigate the long-term effects of macronutrient timing and quality on body composition and metabolic health, and to determine whether reallocating macronutrient intake from dinner to breakfast is associated with healthier body composition and stronger causal inference.

## Data Availability

The datasets from this study are available from the corresponding author upon reasonable request.

## References

[1] Ahmed SK, Mohammed RA. Obesity: Prevalence, causes, consequences, management, preventive strategies and future research directions. Metabol Open. 2025;27:100375. 41041606 10.1016/j.metop.2025.100375 PMC12486175

[2] Europe WHOROf. The challenge of obesity Copenhagen: WHO Regional Office for Europe. [Available from: https://www.who.int/europe/news-room/fact-sheets/item/the-challenge-of-obesity.

[3] Sehgal K, Khanna S. Gut microbiota: a target for intervention in obesity. Expert Rev Gastroenterol Hepatol. 2021;15(10):1169–79. 34329563 10.1080/17474124.2021.1963232

[4] Fink J, Seifert G, Blüher M, Fichtner-Feigl S, Marjanovic G. Obesity Surgery. Dtsch Arztebl Int. 2022;119(5):70–80. 34819222 10.3238/arztebl.m2021.0359 PMC9059860

[5] Caballero B. Humans against Obesity: Who Will Win? Adv Nutr. 2019;10(suppl1):S4–9. 30721956 10.1093/advances/nmy055 PMC6363526

[6] Jafari-Adli S, Jouyandeh Z, Qorbani M, Soroush A, Larijani B, Hasani-Ranjbar S. Prevalence of obesity and overweight in adults and children in Iran; a systematic review. J Diabetes Metab Disord. 2014;13(1):121. 25610814 10.1186/s40200-014-0121-2 PMC4301060

[7] Beulen Y, Martínez-González MA, van de Rest O, Salas-Salvadó J, Sorlí JV, Gómez-Gracia E, et al. Quality of dietary fat intake and body weight and obesity in a Mediterranean population: secondary analyses within the PREDIMED trial. Nutrients. 2018;10(12):2011. 10.3390/nu10122011. 10.3390/nu10122011 PMC6315420 30572588

[8] Goss AM, Goree LL, Ellis AC, Chandler-Laney PC, Casazza K, Lockhart ME, et al. Effects of diet macronutrient composition on body composition and fat distribution during weight maintenance and weight loss. Obesity. 2013;21(6):1139–42. 23671029 10.1002/oby.20191 PMC3735822

[9] Zhao H, Song A, Zheng C, Wang M, Song G. Effects of plant protein and animal protein on lipid profile, body weight and body mass index on patients with hypercholesterolemia: a systematic review and meta-analysis. Acta Diabetol. 2020;57(10):1169–80. 32314018 10.1007/s00592-020-01534-4

[10] Lopes da Silva MV. de Càssia Gonçalves Alfenas R. Effect of the glycemic index on lipid oxidation and body composition. Nutr Hosp. 2011;26(1):48–55. 21519729

[11] Xu F, Greene GW, Earp JE, Adami A, Delmonico MJ, Lofgren IE, et al. Relationships of Physical Activity and Diet Quality with Body Composition and Fat Distribution in US Adults. Obes (Silver Spring). 2020;28(12):2431–40. 10.1002/oby.23018 33099896

[12] Arentson-Lantz EJ, Galvan E, Ellison J, Wacher A, Paddon-Jones D. Improving Dietary Protein Quality Reduces the Negative Effects of Physical Inactivity on Body Composition and Muscle Function. J Gerontol Biol Sci Med Sci. 2019;74(10):1605–11. 10.1093/gerona/glz003 PMC6748768 30689727

[13] van der Merwe C, Münch M, Kruger R. Chronotype differences in body composition, dietary intake and eating behavior outcomes: a scoping systematic review. Adv Nutr. 2022;13(6):2357–405. 10.1093/advances/nmac093. 10.1093/advances/nmac093 PMC9776742 36041181

[14] Goetz AR, Jindal I, Moreno JP, Puyau MR, Adolph AL, Musaad S, et al. The roles of sleep and eating patterns in adiposity gain among preschool-aged children. Am J Clin Nutr. 2022;116(5):1334–42. 10.1093/ajcn/nqac197. 10.1093/ajcn/nqac197 PMC9630867 35833269

[15] Ren X, Yang X, Jiang H, Han T, Sun C. The association of energy and macronutrient intake at dinner vs breakfast with the incidence of type 2 diabetes mellitus in a cohort study: The China Health and Nutrition Survey, 1997–2011. J Diabetes. 2021;13(11):882–92. 33848061 10.1111/1753-0407.13185

[16] Bonnet JP, Cardel MI, Cellini J, Hu FB, Guasch-Ferré M. Breakfast Skipping, Body Composition, and Cardiometabolic Risk: A Systematic Review and Meta-Analysis of Randomized Trials. Obes (Silver Spring Md). 2020;28(6):1098–109. 10.1002/oby.22791 PMC7304383 32304359

[17] Deshmukh-Taskar P, Nicklas TA, Radcliffe JD, O’Neil CE, Liu Y. The relationship of breakfast skipping and type of breakfast consumed with overweight/obesity, abdominal obesity, other cardiometabolic risk factors and the metabolic syndrome in young adults. The National Health and Nutrition Examination Survey (NHANES): 1999–2006. Public Health Nutr. 2013;16(11):2073–82. 23031568 10.1017/S1368980012004296 PMC10271246

[18] Jeans MR, Vandyousefi S, Landry MJ, Leidy HJ, Gray MJ, Bray MS, et al. Breakfast consumption may improve fasting insulin, HOMA-IR, and HbA1c levels in predominately low-income, Hispanic children 7–12 years of age. Nutrients. 2022;14(11):2320. 10.3390/nu1411232. 10.3390/nu14112320 PMC9182585 35684120

[19] Truman SC, Wirth MD, Arp Adams S, Turner-McGrievy GM, Reiss KE, Hébert JR. Meal timing, distribution of macronutrients, and inflammation among African-American women: A cross-sectional study. Chronobiol Int. 2022;39(7):976–83. 35379042 10.1080/07420528.2022.2053702 PMC9177623

[20] Duan D, Pilla SJ, Michalski K, Laferrère B, Clark JM, Maruthur NM. Eatingbreakfast is associated with weight loss during an intensive lifestyleintervention for overweight/obesity. Obesity (Silver Spring). 2022;30:378–88. 10.1002/oby.23340. 10.1002/oby.23340 PMC8820381 35048528

[21] Maukonen M, Kanerva N, Partonen T, Männistö S. Chronotype and energy intake timing in relation to changes in anthropometrics: a 7-year follow-up study in adults. Chronobiol Int. 2019;36(1):27–41. 30212231 10.1080/07420528.2018.1515772

[22] Xiao Q, Garaulet M, Scheer F. Meal timing and obesity: interactions with macronutrient intake and chronotype. Int J Obes (Lond). 2019;43(9):1701–11. 30705391 10.1038/s41366-018-0284-x PMC6669101

[23] Physical status. the use and interpretation of anthropometry. Report of a WHO Expert Committee. World Health Organ Tech Rep Ser. 1995;854:1–452. 8594834

[24] Ashwell M, Gibson S. Waist-to-height ratio as an indicator of ‘early health risk’: simpler and more predictive than using a ‘matrix’ based on BMI and waist circumference. BMJ Open. 2016;6(3):e010159. 26975935 10.1136/bmjopen-2015-010159 PMC4800150

[25] Raymond JL, Morrow K. Krause and Mahan’s Food & the Nutrition Care Process. Elsevier - Health Sciences Division; 2020.

[26] Bazshahi E, Pourreza S, Imani H, Azadbakht L, Ebaditabar M, Davarzani S, et al. The Association of Dietary Energy Density and Body Composition Components in a Sample of Iranian Adults. Front Nutr. 2021;8:751148. 34778343 10.3389/fnut.2021.751148 PMC8588805

[27] Vasheghani-Farahani A, Tahmasbi M, Asheri H, Ashraf H, Nedjat S, Kordi R. The Persian, last 7-day, long form of the International Physical Activity Questionnaire: translation and validation study. Asian J Sports Med. 2011;2(2):106–16. 22375226 10.5812/asjsm.34781 PMC3289200

[28] Fan M, Lyu J, He P. Guidelines for data processing and analysis of the International Physical Activity Questionnaire (IPAQ).2005. Zhonghua liu xing bing xue za zhi = Zhonghua liuxingbingxue zazhi. 2014;35:961-4. http://www.IPAQ.ki.se. 25376692

[29] Horne JA, Ostberg O. A self-assessment questionnaire to determine morningness-eveningness in human circadian rhythms. Int J Chronobiol. 1976;4(2):97–110. 1027738

[30] Chung MH, Chang FM, Yang CC, Kuo TB, Hsu N. Sleep quality and morningness-eveningness of shift nurses. J Clin Nurs. 2009;18(2):279–84. 19120754 10.1111/j.1365-2702.2007.02160.x

[31] Buysse DJ, Reynolds CF 3rd, Monk TH, Berman SR, Kupfer DJ. The Pittsburgh Sleep Quality Index: a new instrument for psychiatric practice and research. Psychiatry Res. 1989;28(2):193–213. 2748771 10.1016/0165-1781(89)90047-4

[32] Salvador Castell G, Serra-Majem L, Ribas-Barba L. What and how much do we eat? 24-hour dietary recall method. Nutr Hosp. 2015;31(Suppl 3):46–8. 25719770 10.3305/nh.2015.31.sup3.8750

[33] Ghafarpour M, Kianfar AH-RH, Ghaffarpour M. A Houshiar Rad The manual for household measures, cooking yields factors and edible portion of food Tehran: Keshaverzi Press; 1999.

[34] Huang TT, Roberts SB, Howarth NC, McCrory MA. Effect of screening out implausible energy intake reports on relationships between diet and BMI. Obes Res. 2005;13(7):1205–17. 16076990 10.1038/oby.2005.143

[35] Kahleova H, Lloren JI, Mashchak A, Hill M, Fraser GE. Meal Frequency and Timing Are Associated with Changes in Body Mass Index in Adventist Health Study 2. J Nutr. 2017;147(9):1722–8. 28701389 10.3945/jn.116.244749 PMC5572489

[36] Leech RM, Worsley A, Timperio A, McNaughton SA. Understanding meal patterns: definitions, methodology and impact on nutrient intake and diet quality. Nutr Res Rev. 2015;28(1):1–21. 25790334 10.1017/S0954422414000262 PMC4501369

[37] Hou W, Gao J, Jiang W, Wei W, Wu H, Zhang Y, et al. Meal Timing of Subtypes of Macronutrients Consumption With Cardiovascular Diseases: NHANES, 2003 to 2016. J Clin Endocrinol Metab. 2021;106(7):e2480–90. 34038544 10.1210/clinem/dgab288

[38] Reynolds A, Mann J, Cummings J, Winter N, Mete E, Te Morenga L. Carbohydrate quality and human health: a series of systematic reviews and meta-analyses. Lancet. 2019;393(10170):434–45. 30638909 10.1016/S0140-6736(18)31809-9

[39] Jenkins DJ, Kendall CW, Augustin LS, Franceschi S, Hamidi M, Marchie A, et al. Glycemic index: overview of implications in health and disease. Am J Clin Nutr. 2002;76(1):s266–73. 10.1093/ajcn/76/1.266S 12081850

[40] Vlahoyiannis A, Andreou E, Aphamis G, Felekkis K, Pieri M, Sakkas GK, et al. Evaluating the evening carbohydrate dilemma: the effect of within-the-day carbohydrate periodization on body composition and physical fitness. Eur J Nutr. 2024;64(1):23. 39585451 10.1007/s00394-024-03540-6

[41] Haldar S, Egli L, De Castro CA, Tay SL, Koh MXN, Darimont C, et al. High or low glycemic index (GI) meals at dinner results in greater postprandial glycemia compared with breakfast: a randomized controlled trial. BMJ Open Diabetes Res Care. 2020;8(1):e001099. 10.1136/bmjdrc-2019-001099. 10.1136/bmjdrc-2019-001099 PMC7202752 32327444

[42] Hou W, Han T, Sun X, Chen Y, Xu J, Wang Y, et al. Relationship Between Carbohydrate Intake (Quantity, Quality, and Time Eaten) and Mortality (Total, Cardiovascular, and Diabetes): Assessment of 2003–2014 National Health and Nutrition Examination Survey Participants. Diabetes Care. 2022;45(12):3024–31. 36174119 10.2337/dc22-0462

[43] Muñoz-Cabrejas A, Laclaustra M, Guallar-Castillón P, Casasnovas JA, Marco-Benedí V, Calvo-Galiano N, et al. Low-Quality Carbohydrate Intake Is Associated With a Higher Prevalence of Metabolic Syndrome: The AWHS Study. J Clin Endocrinol Metab. 2024;109(9):e1768–75. 38141071 10.1210/clinem/dgad706

[44] Poggiogalle E, Jamshed H, Peterson CM. Circadian regulation of glucose, lipid, and energy metabolism in humans. Metabolism. 2018;84:11–27. 29195759 10.1016/j.metabol.2017.11.017 PMC5995632

[45] Zhao L, Hutchison AT, Heilbronn LK. Carbohydrate intake and circadian synchronicity in the regulation of glucose homeostasis. Curr Opin Clin Nutr Metab Care. 2021;24(4):342–8. 33883418 10.1097/MCO.0000000000000756

[46] Wefers J, van Moorsel D, Hansen J, Connell NJ, Havekes B, Hoeks J, et al. Circadian misalignment induces fatty acid metabolism gene profiles and compromises insulin sensitivity in human skeletal muscle. Proc Natl Acad Sci U S A. 2018;115(30):7789–94. 29987027 10.1073/pnas.1722295115 PMC6065021

[47] Phillips CM, Kesse-Guyot E, McManus R, Hercberg S, Lairon D, Planells R, et al. High Dietary Saturated Fat Intake Accentuates Obesity Risk Associated with the Fat Mass and Obesity-Associated Gene in Adults. J Nutr. 2012;142(5):824–31. 22457394 10.3945/jn.111.153460

[48] von Frankenberg AD, Marina A, Song X, Callahan HS, Kratz M, Utzschneider KM. A high-fat, high-saturated fat diet decreases insulin sensitivity without changing intra-abdominal fat in weight-stable overweight and obese adults. Eur J Nutr. 2017;56(1):431–43. 26615402 10.1007/s00394-015-1108-6 PMC5291812

[49] Kim S-M, Neuendorff N, Chapkin RS, Earnest DJ. Role of Inflammatory Signaling in the Differential Effects of Saturated and Poly-unsaturated Fatty Acids on Peripheral Circadian Clocks. EBioMedicine. 2016;7:100–11. 27322464 10.1016/j.ebiom.2016.03.037 PMC4913702

[50] Piers LS, Walker KZ, Stoney RM, Soares MJ, O’Dea K. Substitution of saturated with monounsaturated fat in a 4-week diet affects body weight and composition of overweight and obese men. Br J Nutr. 2003;90(3):717–27. 13129479 10.1079/bjn2003948

[51] Lim MT, Pan BJ, Toh DWK, Sutanto CN, Kim JE. Animal protein versus plant protein in supporting lean mass and muscle strength: a systematic review and meta-analysis of randomized controlled trials. Nutrients. 2021;13(2):661. 10.3390/nu13020661. 10.3390/nu13020661 PMC7926405 33670701

[52] Berrazaga I, Micard V, Gueugneau M, Walrand S. The role of the anabolic properties of plant- versus animal-based protein sources in supporting muscle mass maintenance: a critical review. Nutrients. 2019;11(8):1825. 10.3390/nu11081825. 10.3390/nu11081825 PMC6723444 31394788

[53] van Vliet S, Burd NA, van Loon LJ. The Skeletal Muscle Anabolic Response to Plant- versus Animal-Based Protein Consumption. J Nutr. 2015;145(9):1981–91. 26224750 10.3945/jn.114.204305

[54] Gorissen SHM, Witard OC. Characterising the muscle anabolic potential of dairy, meat and plant-based protein sources in older adults. Proc Nutr Soc. 2018;77(1):20–31. 28847314 10.1017/S002966511700194X

[55] Wirth J, Hillesheim E, Brennan L. The Role of Protein Intake and its Timing on Body Composition and Muscle Function in Healthy Adults: A Systematic Review and Meta-Analysis of Randomized Controlled Trials. J Nutr. 2020;150(6):1443–60. 32232404 10.1093/jn/nxaa049

[56] Berryman CE, Lieberman HR, Fulgoni VL, Pasiakos SM. Greater protein intake at breakfast or as snacks and less at dinner is associated with cardiometabolic health in adults. Clin Nutr. 2021;40(6):4301–8. 33583662 10.1016/j.clnu.2021.01.018

[57] Richter CK, Skulas-Ray AC, Champagne CM, Kris-Etherton PM. Plant protein and animal proteins: do they differentially affect cardiovascular disease risk? Adv Nutr. 2015;6(6):712–28. 26567196 10.3945/an.115.009654 PMC4642426

[58] Mariotti F. Animal and Plant Protein Sources and Cardiometabolic Health. Adv Nutr. 2019;10(Suppl4):S351–66. 31728490 10.1093/advances/nmy110 PMC6855969

[59] Anderson KE, Rosner W, Khan MS, New MI, Pang S, Wissel PS, et al. Diet-hormone interactions: Protein/carbohydrate ratio alters reciprocally the plasma levels of testosterone and cortisol and their respective binding globulins in man. Life Sci. 1987;40(18):1761–8. 3573976 10.1016/0024-3205(87)90086-5

[60] Drummond MJ, Rasmussen BB. Leucine-enriched nutrients and the regulation of mammalian target of rapamycin signalling and human skeletal muscle protein synthesis. Curr Opin Clin Nutr Metab Care. 2008;11(3):222–6. 18403916 10.1097/MCO.0b013e3282fa17fb PMC5096790

[61] Story JA. The Role of Dietary Fiber in Lipid Metabolism. In: Paoletti R, Kritchevsky D, editors. Advances in Lipid Research. Volume 18. Elsevier; 1981. 229–46. 10.1016/b978-0-12-024918-3.50012-5 6275660

[62] Hegde A, Roberts A. Is plant protein equally as effective at promoting lean muscle mass compared to meat? J Stud Res. 2023;11:1–15. 10.47611/jsrhs.v11i3.3487.

[63] Ali M, Reutrakul S, Petersen G, Knutson KL Associations between timing and duration of eating and glucose metabolism: a nationally representative study in the U.S. Nutrients. 2023;15(3):729. 10.3390/nu15030729. 36771435 10.3390/nu15030729 PMC9919634

[64] Rangaraj VR, Siddula A, Burgess HJ, Pannain S, Knutson KL. Association between timing of energy intake and insulin sensitivity: a cross-sectional study. Nutrients. 2020 Feb 16;12(2):503. doi:10.3390/nu12020503. 10.3390/nu12020503 PMC7071301 32079066

[65] Richter J, Herzog N, Janka S, Baumann T, Kistenmacher A, Oltmanns KM. Twice as high diet-induced thermogenesis after breakfast vs dinner on high-calorie as well as low-calorie meals. J Clin Endocrinol Metab. 2020 Mar 1;105(3):dgz311. doi:10.1210/clinem/dgz311. 10.1210/clinem/dgz311 32073608

[66] Ni Y, Wu L, Jiang J, Yang T, Wang Z, Ma L, et al. Late-Night Eating-Induced Physiological Dysregulation and Circadian Misalignment Are Accompanied by Microbial Dysbiosis. Mol Nutr Food Res. 2019;63(24):1900867. 10.1002/mnfr.201900867 31628714

[67] Kim YI, Kim E, Lee Y, Park J. Role of late-night eating in circadian disruption and depression: a review of emotional health impacts. Phys Act Nutr. 2025;29(1):18–24. 40443246 10.20463/pan.2025.0003 PMC12127805

[68] Leung GKW, Huggins CE, Ware RS, Bonham MP. Time of day difference in postprandial glucose and insulin responses: Systematic review and meta-analysis of acute postprandial studies. Chronobiol Int. 2020;37(3):311–26. 31782659 10.1080/07420528.2019.1683856

[69] Carabuena TJ, Boege HL, Bhatti MZ, Whyte KJ, Cheng B, St-Onge MP. Delaying mealtimes reduces fat oxidation: A randomized, crossover, controlled feeding study. Obes (Silver Spring). 2022;30(12):2386–95. 10.1002/oby.23566 PMC9691571 36238978

[70] Kelly KP, McGuinness OP, Buchowski M, Hughey JJ, Chen H, Powers J, et al. Eating breakfast and avoiding late-evening snacking sustains lipid oxidation. PLoS Biol. 2020;18(2):e3000622. 32108181 10.1371/journal.pbio.3000622 PMC7046182

[71] Jeong S, Lee H, Jung S, Kim JY, Park S. Higher energy consumption in the evening is associated with increased odds of obesity and metabolic syndrome: findings from the 2016–2018 Korea National Health and Nutrition Examination Survey (7th KNHANES). Epidemiol Health. 2023;45:e2023087. 37752794 10.4178/epih.e2023087 PMC10867517

[72] Hermenegildo-López Y, Donat-Vargas C, Sandoval-Insausti H, Moreno-Franco B, Rodríguez-Ayala M, Rey-García J, et al. A higher intake of energy at dinner is associated with incident metabolic syndrome: a prospective cohort study in older adults. Nutrients. 2021;13(9):3035. 10.3390/nu13093035. 10.3390/nu13093035 PMC8465293 34578912

[73] McHill AW, Phillips AJ, Czeisler CA, Keating L, Yee K, Barger LK, et al. Later circadian timing of food intake is associated with increased body fat. Am J Clin Nutr. 2017;106(5):1213–9. 28877894 10.3945/ajcn.117.161588 PMC5657289

[74] BaHammam AS, Pirzada A. Timing Matters: The Interplay between Early Mealtime, Circadian Rhythms, Gene Expression, Circadian Hormones, and Metabolism-A Narrative Review. Clocks Sleep. 2023;5(3):507–35. 37754352 10.3390/clockssleep5030034 PMC10528427

